# The forgotten spine score: study protocol for a novel patient-centered outcome measure in lumbar spine fusion surgery

**DOI:** 10.3389/fsurg.2025.1547829

**Published:** 2025-05-22

**Authors:** Luca Ambrosio, Jordy Schol, Yuto Otani, Giorgia Petrucci, Elisabetta de Rinaldis, Shota Tamagawa, Fabrizio Russo, Rocco Papalia, Daisuke Sakai, Gianluca Vadalà, Vincenzo Denaro

**Affiliations:** ^1^ Operative Research Unit of Orthopaedic and Trauma Surgery, Fondazione Policlinico Universitario Campus Bio-Medico, Rome, Italy; ^2^ Research Unit of Orthopaedic and Trauma Surgery, Department of Medicine and Surgery, Università Campus, Bio-Medico di Roma, Rome, Italy; ^3^ Department of Orthopaedic Surgery, Tokai University School of Medicine, Isehara, Japan; ^4^ Department of Medicine for Orthopaedics and Motor Organ, Juntendo University Graduate School of Medicine, Tokyo, Japan; ^5^ Department of Orthopaedics, Faculty of Medicine, Juntendo University, Tokyo, Japan

**Keywords:** low back pain, patient-reported outcome measures, disability, spinal fusion, disc degeneration, spine surgery, lumbar spine

## Abstract

Lumbar spine fusion (LSF) surgeries have increased due to the rising prevalence of degenerative spine disorders and advancements in surgical techniques. However, the success of these procedures should be evaluated from the patient's perspective, emphasizing the need for effective patient-reported outcome measures (PROMs). Existing PROMs often focus on symptoms and lack specific validation for surgical patients undergoing LSF. Inspired by the Forgotten Joint Score used in joint replacement surgery, here we introduce the Forgotten Spine Score (FSS), a new patient-centered PROM designed to assess the extent to which patients “forget” about the operated spinal segment(s) in their daily lives. This study protocol outlines our approach to develop and validate the FSS as a reliable tool for evaluating outcomes in LSF patients, focusing on their perception of surgery's impact on daily life and well-being. This multi-institutional cross-sectional study will involve a pilot phase to refine the newly designed 18-item FSS survey in Italian and Japanese, followed by a validation phase to assess the adjusted survey in the respective populations. Eligible participants will include adults who have undergone LSF for degenerative disorders at varying follow-up times. The FSS will be administered alongside the Oswestry Disability Index (ODI). Statistical analyses will assess internal consistency, reliability and validity, with significance set at *p* < 0.05. The survey will be assessed based on its correlation with the ODI, potential for enhanced sensitivity, and overall appreciation among LSF patients. By improving our understanding of patient-centered outcomes, the FSS has the potential to inform clinical decision-making and patient care.

## 1 Introduction

Over the past few decades, there has been a remarkable surge in lumbar spine fusion (LSF) surgeries globally. This escalation can be attributed to the rising prevalence of degenerative spine disorders, the integration of innovative surgical implants, and the development of minimally invasive techniques (1, 2). Despite advances in surgical technology and methodology, the ultimate success of LSF procedures remains inherently linked to the subjective perspective of patients. Recognizing this, the routine incorporation of patient-reported outcome measures (PROMs) has become crucial in bridging the gap between clinicians' and patients' perceptions of clinical reality (3, 4). By integrating PROMs into clinical practice, healthcare providers can tailor treatment strategies that align with patients' preferences and needs.

To date, PROMs have become universally acknowledged as indispensable tools in research, clinical decision-making, patient-centered care, health policy, and even reimbursement schemes (3). Since the introduction of the Oswestry Disability Index (ODI) in 1980 (5, 6), PROMs have gained increasing popularity in spine care, with over 200 different scores described to date. However, a concerning aspect is that up to 30% of these tools have been presented in studies characterized by low quality and insufficient formal validation (3, 7). This lack of standardization, combined with similarities among different tools, contributes to ambiguity in selecting the most appropriate scale, which may deter care providers from routinely implementing PROMs in clinical practice (8).

As most PROMs for individuals with spinal disorders are symptom- or disease-centered, they often overlook the unique patient perspective on clinical success (9). In contrast, Behrend et al. introduced a novel PROM for hip and knee arthroplasty that emphasizes the patient's ability to forget about the artificial joint in everyday life as the ultimate goal of joint replacement surgery (10). This concept recognizes that the awareness of an operated joint carries a negative connotation; healthy joints typically do not prompt awareness and are essentially “forgotten” (10). The ability to “forget” about the operated joint implies several beneficial outcomes experienced in daily life, including pain relief, reduced disability, restored range of motion, and decreased social anxiety associated with, for example, visible scarring. In just over a decade, the Forgotten Joint Score has been translated into 28 languages and utilized in more than 350 publications (11).

Recognizing the epidemiologic, clinical, and socioeconomic parallels between joint replacement and LSF, we advocate for a similarly disruptive, patient-centered approach to developing a new PROM for assessing clinical outcomes in patients undergoing LSF: the “Forgotten Spine Score” (FSS). This novel survey is designed to reflect the patient's ability to live their daily life without being constantly aware of operated spinal segments, effectively “forgetting” their fused spine. Unlike commonly used PROMs in the lumbar spine field, such as the ODI (5, 6) and Roland Morris Disability Questionnaire (12), which primarily focus on symptoms and disease, our new survey emphasizes the patient's ability to engage in everyday activities. Furthermore, it has been designed to minimize the use of technical language, promoting ease of understanding and usability, particularly for those with more limited health literacy (13, 14), to reduce potential bias.

The primary objective of this protocol is to outline the procedure for developing and validating the “Forgotten Spine Score” survey as a novel, patient-friendly PROM dedicated to assessing outcomes following LSF. By shifting the focus from the disease to the patient, we aim to enhance the patient-physician relationship, enabling a more personalized follow-up approach that captures the aspects of patients' lives that truly matter to them.

## 2 Methods and analysis

### 2.1 Design and setting

**Figure 1:**
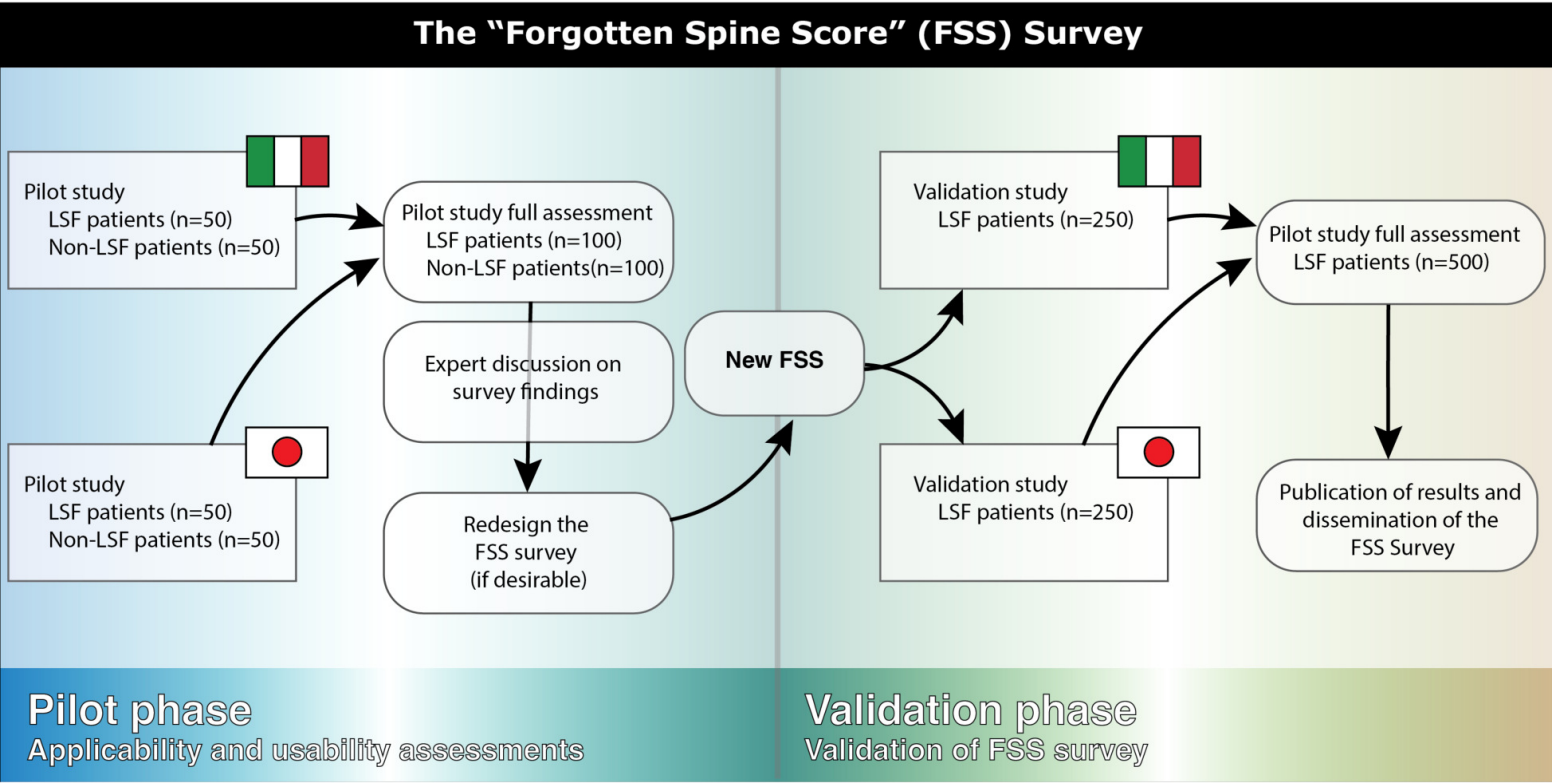
Flow-chart representing the organization of the proposed cross-sectional study.

This study is designed as a multi-institutional cross-sectional study which will be conducted in Italian at Campus Bio-Medico University of Rome (Rome, Italy) and in Japanese at Tokai University School of Medicine (Isehara, Japan) and Juntendo University Hospital (Tokyo, Japan). The study will be divided into two phases (Figure 1):

1. Pilot Phase: A preliminary study will be carried out at both institutions to assess the survey's initial version for clarity and relevance. Feedback gathered from participants during this phase will help refine and finalize the survey tool, ensuring it is well-understood and applicable.
2. Final Phase: Following the pilot phase, a larger cross-sectional study will be conducted to fully validate the adjusted survey in both the Italian and Japanese populations.

### 2.2 Questionnaire development

The Italian and Japanese translations of the FSS survey were developed through direct translation of an initial English version, following the Principles of Good Practice for the Translation and Cultural Adaptation Process for Patient-Reported Outcomes Measures from the ISPOR (15). In brief, after initial preparation, two independent forward translations (Italian and Japanese) were produced by in-country experts within a multidisciplinary team including clinicians, study nurses, researchers, and a biostatistician, drawing on available evidence and clinical experience. After careful reconciliation, a back translation into English was conducted to assess conceptual equivalence between the two languages. Subsequent critical discussions among the authors from Italy and Japan led to the harmonization of the survey, resulting in an 18-item questionnaire. To incorporate patient perspectives and conduct cognitive debriefing, a small group of patients who had undergone LSF were interviewed during the pilot phase, focusing on the impact of surgery on their daily lives and social interactions. The Italian and Japanese versions can be found as Supplementary Materials. Based on the findings from the pilot phase, the cognitive debriefing process will be reviewed, and the final version of the FSS will be proofread and finalized before distribution to patients in the validation phase.

The questionnaire items have been designed to assess patients' awareness of their fused spinal segment during various daily activities and social interactions, as outlined in the following questions and explanations:

Are you aware of your fused spine when…

1. …you are lying down?
2. …you are sitting for longer than 15 min?
3. …you are standing for longer than 15 min?
4. …you are climbing the stairs?
5. …you are washing and dressing yourself?
6. …you are walking or hiking?
7. …you are driving a car?

Are you aware of your fused spine when…

8. …you are doing household activities? (for example, cleaning, gardening, taking care of kids)
9. …you are bending down to put on socks or tie your shoes?
10. …you are picking something up from the floor?
11. …you standing up from a seated or lying position?

Are you aware of your fused spinal section when…

12. … you are walking with a bag or backpack?
13. … you are lifting heavy (>10 kg) objects (for example, a vacuum cleaner, packet of water bottles, kids, luggage, etc.)?
14. … you are holding/carrying something of medium weight (>2 kg) for more than 15 min (for example, groceries bags, laptop, books, etc.)?

Has your fused spine made you refrain from participating in…

15. … activities that may show surgical scars (such as during swimming)?
16. … social events and parties?
17. … sports activities?
18. … sex or intimate activities?

Furthermore, the items are categorized into four subscales:

• General Awareness (items 1–7)
• Bending and Twisting Activities (items 8–11)
• Weight-Carrying Activities (items 12–14)
• Social Activities (items 15–18)

Each item will be scored on a scale of 0 (never) to 4 (always), with an additional option of “does not apply to me” (N/A). A full worksheet of this survey can be found as Supplementary Item S1. The final score will be computed using the formula:

$$FSS=\frac{Total\;score}{4*(18-[Number\;of\;\;\prime \prime N/A\prime \prime \;\;responses])}*100$$

The formula will give a score from 0% to 100%, where a higher score signifies a respondent that is more aware of their fused spine in their life. The formula is also designed to correct for potential unapplicable situation, for example patients not partaking in sports or not driving a car. To address potential oversights in the questionnaire items, an open-ended question will be included in the pilot phase, inviting participants to suggest additional items or modifications. Additionally, participants are also prompted to score the usefulness of the survey according to the following Likert scale: “Very useful” (1), “Useful” (2), “Not useful” (3), “Not useful at all” (4), and “no opinion” (N/A). The FSS survey is anticipated to require only a few minutes for participants to complete. When combined with the ODI survey and consent forms, the total time for completion is expected to range from 5 to 10 min.

### 2.3 Participant recruitment

Patients that have undergone LSF due to degenerative disorders (e.g., degenerative spondylolisthesis, spinal stenosis, degenerative scoliosis) at Fondazione Policlinico Universitario Campus Bio-Medico (Rome, Italy), Tokai University Hospital (Isehara, Japan), and Juntendo University Hospital (Tokyo, Japan) will be eligible for enrollment and will be contacted by clinical and/or administrative staff. Inclusion criteria specify patients aged 18 years or older who have had single- or multi-level LSF between the L1 and S1. Exclusion criteria include revision surgeries, LSF performed for reasons such as trauma, infection, or cancer, as well as cases where the fusion extends into the thoracic spine or pelvis. Additionally, individuals who cannot comprehend Italian or Japanese, depending on the institution, will be excluded from the study. Data collection will cover a range of sociodemographic and clinical information, including age, sex, education level, date and type of LSF, number of fused levels, type of surgical approaches (e.g., lumbar lateral interbody fusion, posterior lumbar interbody fusion, transforaminal lumbar interbody fusion), and time since surgery.

During the pilot phase, a minimum of 50 LSF patients will be recruited for the Italian survey and 50 for the Japanese survey. Additionally, a matched group of 50 “healthy” volunteers who have not undergone LSF surgery will be included at each institution to establish reference values for the FSS. For the second phase of the study, aimed at validating both surveys, at least 250 LSF participants will be recruited at each institution in both countries. The details of sample size calculations have been outlined below.

### 2.4 Questionnaire administration

The FSS survey will be administered during one of the follow-up outpatient visits after LFS. In addition to the FSS, participants will complete the Italian (16) or Japanese (17) version of the ODI (5, 6). The ODI is a well-established and validated PROM that assesses disability related to various daily activities and will serve as a reference for evaluating the FSS results. Participants will have the option to complete either a paper-based version of the questionnaire or a web-based version via Google Forms at the discretion of the healthcare provider.

Patient recruitment will occur concurrently across institutions until the desired sample size is achieved for both language versions (Italian and Japanese). The pilot study is expected to be completed within six months, with the validation study commencing thereafter, following the refinement of the FSS based on pilot outcomes and expert discussions with patients and investigators. Special attention will be given to internal consistency reliability and inter-item correlation, particularly in relation to aligning categories with ODI outcomes and within FSS subscales. We will also assess potential ceiling and floor effects, which may indicate a need for survey revisions. Additionally, we will carefully track the number of “N/A” responses for specific questions to evaluate their applicability to LSF patients and general merit. Finally, qualitative feedback from patients and other participants will be thoroughly reviewed, and the survey design or wording may be altered based on this input. The revised survey will then undergo a final validation study, projected to conclude within a 12-month timeframe.

### 2.5 Sample size calculation

Sample sizes for both the pilot and validation populations have been estimated based on previous studies validating the Forgotten Joint Score (10) and other relevant PROMs in lumbar spine research. Notably, among the top 10 most frequently cited PROMs mentioned earlier, the majority did not incorporate a pilot phase. For those that did, the recruited participants ranged from 34 to 39 individuals. In contrast, the average number of participants in the validation population across these studies was 229 participants (18). In addition to this literature-based rationale, we also conducted a formal sample size estimation to ensure adequate statistical power for the validation of the newly developed FSS survey. Specifically, to assess the validity of the FSS survey by correlating it with ODI outcomes, we assumed a two-sided alpha of 0.05 and a desired power of 80%. Under these assumptions, a required sample size of 29 participants was estimated for detecting a moderate expected correlation (*r* = 0.5), and up to 194 participants for detecting a mild correlation (*r* = 0.2). To account for potential dropouts, variability, and lower-than-expected correlations, we set a minimum target of 50 participants for a moderate or 250 participants for a mild correlation. Therefore, our pilot study aims to include at least 50 participants, while the validation study will target a minimum of 250 participants. Furthermore, our study will be conducted in duplicate, both in Japan and Italy, further enhancing the robustness of our study and its potential findings.

### 2.6 Statistical analysis

Depending on data distribution, descriptive statistics will summarize continuous variables as mean ± standard deviation or median and interquartile range. Normality will be assessed using the Shapiro–Wilk test. Categorical data will be expressed as absolute (n) and relative (%) frequencies.

During the pilot study, item selection will involve several analytical steps to ensure the survey's reliability and validity. Cronbach's alpha will be calculated to assess internal consistency reliability of the FSS items. A Cronbach's alpha value of 0.70 or higher will be considered acceptable, indicating that the items measure a unidimensional construct. If the value falls below this threshold, specific items may be evaluated for potential revision or removal based on their inter-item correlations, ensuring that each item contributes meaningfully to the overall scale. The percentage of “N/A” responses and missing items will be analyzed to assess the completeness and relevance of each item. Items with a high rate of missing or “N/A” responses—defined as exceeding 20%—will be flagged for potential revision, as this may indicate issues with clarity, relevance, or applicability to the LSF patient population. However, decisions regarding item revision will not be based solely on quantitative thresholds. Instead, each flagged item will be carefully reviewed by the research team, in consultation with patient feedback where available. For example, certain items, such as those related to driving, may exhibit a high proportion of “N/A” responses simply because not all patients drive, yet these items may still be considered clinically important for a subset of participants. Final decisions regarding item retention, modification, or removal will be made during expert panel discussions to ensure that the final version of the survey remains both comprehensive and applicable to the intended patient population. In the validation phase, all participants will initially be included in the primary analyses, regardless of missing or “N/A” responses. In addition, a sensitivity analysis will be conducted by excluding participants with more than 30% missing or “N/A” responses to evaluate the potential impact of incomplete data on the results. This approach will allow us to assess the robustness of the findings and determine whether missing data significantly influence the survey's performance.

For the final analysis in the validation study, the Italian and Japanese versions will be assessed both as independent surveys and as combined cohorts. The assessments aim to determine differences in responses between the outcomes derived from both translated surveys and to contextualize these differences within cultural disparities or potential variations in medical practice. The frequency of response categories will be evaluated to identify patterns in how participants respond to each item. This analysis will help determine whether certain response options are underutilized or if adjustments are needed to the scale to capture a broader range of participant experiences. Ceiling and floor effects of the FSS will be assessed by calculating the percentage of participants who achieve the highest or lowest possible scores, respectively. A significant ceiling effect occurs when a large proportion of 15% or more of respondents score at the upper limit of the scale, indicating that the survey may not be sensitive enough to detect variations in higher-functioning individuals. Conversely, a floor effect, where more than 15% participants score at the lower limit, suggests that the survey may not adequately capture the experiences of those with more severe limitations. Evaluating these effects is crucial for refining the FSS to ensure it captures the full spectrum of functional status.

A *p*-value of less than 0.05 will be considered statistically significant. Data collection and initial processing will be done using Microsoft Excel (version 16.92 or above, Microsoft, USA), while further analysis and graphical illustrations will be performed using GraphPad Prism for Mac (version 10.4.1 or above, GraphPad Software LLC, USA).

Construct validity will be evaluated by comparing responses from patients with healthy controls using independent-sample Student's *T*-test or Mann–Whitney *U*-test, as appropriate. Convergent validity will be assessed through correlation analyses between the FSS and ODI scores, as well as correlations with time since surgery, number of fused levels, and other sociodemographic variables, especially related to age and sex of the participants.

## 3 Discussion

This study protocol outlines the development and validation of the FSS, a novel patient-centered PROM designed to assess the extent to which patients “forget” about their fused spine segments in daily life following LSF surgery. By adopting a patient-centered approach, this study represents a significant advancement in addressing the limitations of existing PROMs in spine surgery research.

**Figure 2:**
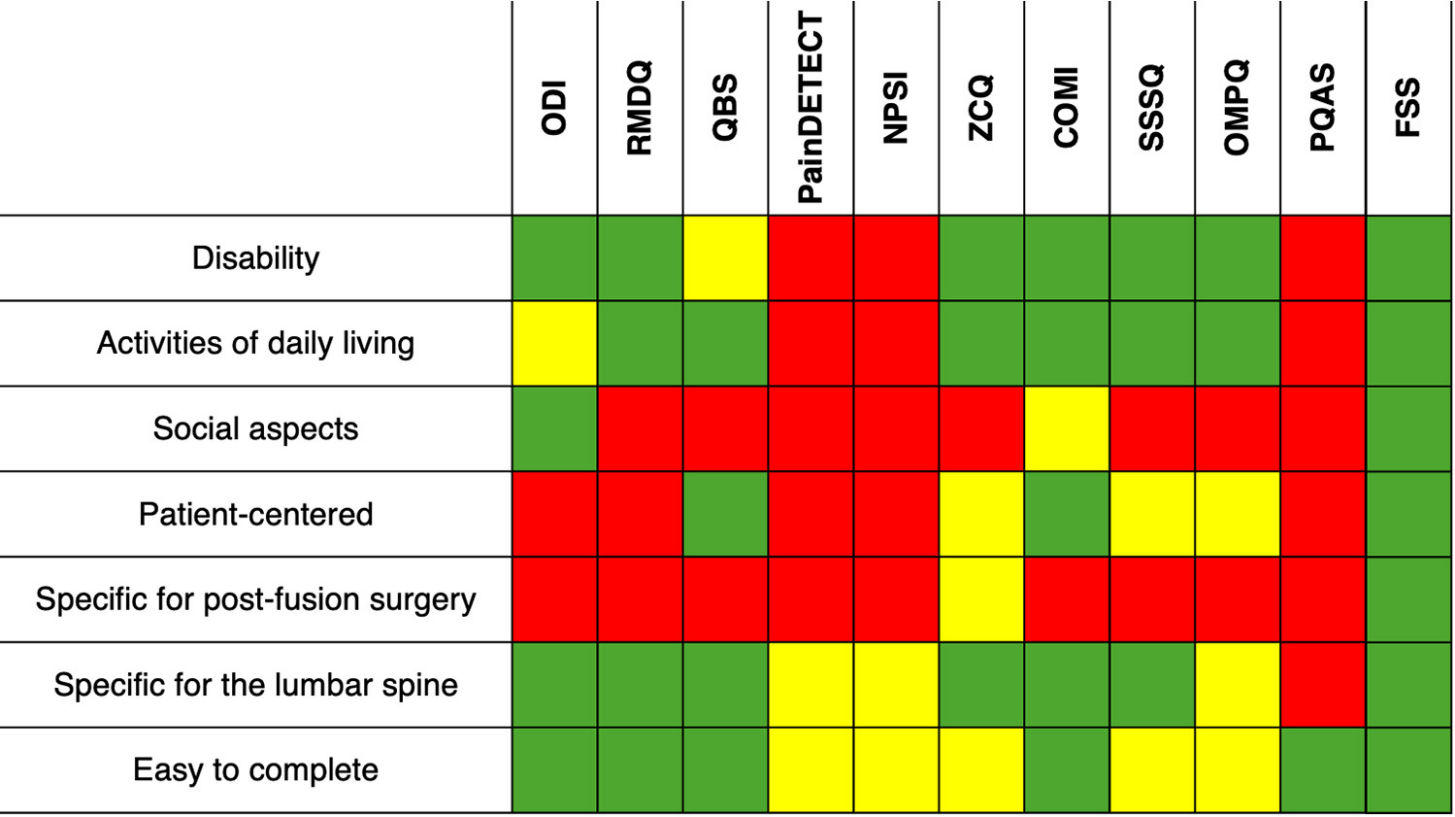
Comparative applicability of various items (left side) to the top 10 cited PROMs in the lumbar spine field and the newly proposed forgotten spine score (FSS, right side). Green denotes complete applicability, yellow indicates partial applicability, and red signifies incomplete applicability. COMI, core outcome measures index; NPSI, neuropathic pain symptom inventory; ODI, oswestry disability index; OMPQ, orebro musculoskeletal pain questionnaire; QBS, Quebec back scale; PQAS, pain quality assessment scale; RMDQ, roland morris disability questionnaire; SSSQ, Swiss spinal stenosis questionnaire; ZCQ, Zurich claudication questionnaire.

The ten most cited PROMs in the lumbar spine field, i.e., the ODI, Roland Morris Disability Questionnaire (RMDQ), Quebec Back Scale (QBS), PainDETECT, Neuropathic Pain Symptom Inventory (NPSI), Zurich Claudication Questionnaire (ZCQ), Core Outcome Measures Index (COMI), Swiss Spinal Stenosis Questionnaire (SSSQ), Orebro Musculoskeletal Pain Questionnaire (OMPQ), and Pain Quality Assessment Scale (PQAS), are predominantly symptom- or disease-centered (4) (Figure 2). Indeed, very few delve into the social aspects and subjective feelings of patients. Furthermore, these commonly employed scales often feature numerous items and complex language, rendering them cumbersome for patients to complete and increasing the risk of response bias. Notably, none of these scales are specifically tailored for LSF patients, and some are not even specific to the lumbar spine. Considering that most of these PROMs were introduced between 15 and 40 years ago and that outcomes following LSF have significantly improved, it is likely that classical scales might be unable to detect subtle clinical differences due to floor- and ceiling effects.

One of the key strengths of this study is the specific personal emphasis of the FSS, which prioritizes real-world patient experiences and focuses on the extent to which patients regain a sense of normalcy post-surgery. The concept of “forgetting” the operated spine has the potential to redefine clinical success, shifting away from symptom-based evaluations to a holistic understanding of recovery. According to Behrend et al., this novel approach is able integrate a variety of variables such as pain, stiffness, function in activities of daily living, patients' expectations, activity levels, and psychosocial factors (10). Additionally, the multi-center design enhances the generalizability of the findings. By including diverse populations across Italy and Japan, the study captures cultural and contextual variations, ensuring that the FSS is adaptable and applicable to broader patient populations. Care must be taken to ensure that all questions are culturally appropriate in both nations, with consideration for the future development of alternative translations of the FSS survey, including English. The rigorous sample selection process, incorporating well-defined inclusion and exclusion criteria, minimizes bias and ensures the validity of the outcomes.

However, this study is not without limitations. The recruitment process may introduce selection bias, as participants who will consent to the study may not represent the full spectrum of LSF patients. For instance, individuals with more severe functional limitations or those with lower health literacy may be underrepresented. This limitation could affect the translatability of the findings to other cohort specifications. Furthermore, the self-reported nature of PROMs, including the FSS, is inherently subject to recall and response biases, such as social desirability bias, which may influence the accuracy of the data collected. To help mitigate these biases, the FSS survey instructions explicitly state that responses are anonymous and emphasize that honest reporting is essential. It is also important to note that the FSS does not directly ask patients whether they have “forgotten” their spine but rather focuses on their awareness of their fused spine during daily activities, serving as an indirect indicator of functional integration. Additionally, the FSS is designed to complement, rather than replace, objective clinical outcomes such as return-to-work status or radiological assessments, offering an additional patient-centered perspective. The two-phase design, involving a pilot and validation phase, ensures thorough refinement and testing of the FSS. Nonetheless, the iterative process may lead to prolonged timelines for full implementation and adoption of the tool in clinical practice. Despite these limitations, the FSS represents a significant step forward in promoting patient-centered care in spine surgery. By providing clinicians with a validated tool to assess outcomes that matter most to patients, the FSS can inform personalized treatment plans and enhance patient-physician communication. Additionally, the simplicity and brevity of the FSS make it a practical choice for routine clinical use, potentially encouraging its widespread adoption. Future studies should focus on validating the FSS in additional languages and populations to further establish its global applicability. Currently the FSS is specifically designed for patients undergoing LSF. However, its inherent versatility suggests potential for adaptation to other spine surgical populations, such as those undergoing anterior cervical discectomy and fusion or posterior cervical spine fusion. Additionally, this study is intended to solely validate the questionnaire and will not evaluate the capacity of the FSS to capture outcome changes with time. Longitudinal studies are needed to evaluate its sensitivity to changes across various follow-up periods, providing insights into its utility for monitoring long-term outcomes. Additionally, comparative studies with other PROMs could highlight the FSS's unique strengths and areas for further refinement.

## 4 Ethics and dissemination

### 4.1 Ethical considerations

Ethical approvals have been obtained from the Institutional Review Board of the Campus Bio-Medico University of Rome (Approval No. 301.24 CET2) and the Ethics Committees of Tokai University Hospital (Approval No. 24R106-001H) and Juntendo University Hospital (Approval No. E24-0399). The study will adhere to the principles outlined in the Declaration of Helsinki, along with any applicable local regulatory requirements. Informed consent will be obtained from all participants prior to the administration of the survey package. To safeguard participant privacy, the study will not collect any sensitive information, such as details regarding racial or ethnic background, sexual orientation, or religious beliefs. All data will be anonymized at the point of collection. Participation is strictly voluntary, and individuals will be required to give informed consent before they are enrolled. Throughout the course of the study, the research team is dedicated to maintaining the highest standards of ethical conduct and legal compliance.

### 4.2 Safety considerations

There are no significant health related risks associated with our proposed study. We will ensure patient data remains confidential and no names, identifying materials, or images of participants will be made public to ensure the privacy of all participants in our cross-sectional study.

### 4.3 Dissemination plan

The findings of this study will be disseminated through various avenues to maximize visibility and impact. We plan to publish the results in a peer-reviewed journal, particularly those specializing in spinal surgery and rehabilitation. Additionally, we will present our results at relevant national and international conferences, including the International Society for the Study of the Lumbar Spine (ISSLS), to foster discussion and collaboration with other researchers and clinicians in the field. All data associated with the validation study will be made publicly accessible upon publication. We will also explore the creation of summaries or infographics to effectively communicate key findings to a broader audience, including healthcare stakeholders, patient advocacy groups, and policymakers. Furthermore, the final versions of the Italian and Japanese surveys will be made publicly available at no cost, provided that appropriate references to our work are acknowledged when they are utilized. If the study results lay the groundwork for a larger-scale investigation, we aim to validate the English version of the survey. To achieve this, we will seek the support and participation of a global spine surgery society or spine patient group to implement and validate the English version of the survey within predominantly English-speaking communities or other languages if desired.

In conclusion, the development of the FSS aligns with the growing emphasis on patient-centered outcome measures in spine surgery. By addressing gaps in existing PROMs and focusing on patients' real-world experiences, this study has the potential to redefine how success is measured in lumbar spine fusion surgery.
