# Supplementary material for: The forgotten spine score: study protocol for a novel patient-centered outcome measure in lumbar spine fusion surgery
Source: Front Surg. 2025 May 22;12:1547829. doi: 10.3389/fsurg.2025.1547829 (PMC12231350; doi:10.3389/fsurg.2025.1547829)
Supplement: Supplementary file 1 [file Datasheet1.pdf]

TOKAI UNIVERSITY

この度はアンケートにご協力いただき、ありがとうございます。

現在、脊椎固定術（融合術）が患者様の日常生活にどのような影響を与えるか、そして手術が患者様の視点から見て効果的かどうかをより深く確認するため、アンケートへのご協力をお願いしております。

ここでは、３種類のアンケートにお答えいただきます。

1. 「ODI アンケート」
   10 の簡単な質問からなるアンケートです。

2. 「FSS アンケート」
   18 の簡単な質問からなるアンケートです。

3. 上記２つのアンケートに対する感想

皆様のご意見は、この研究にとって非常に貴重かつ重要であり、今後脊椎手術を受ける患者様に提供する医療の質を向上させる可能性があります。
どうぞ遠慮なく正直にお答えください。
なお、ご回答はプライバシーを厳守し、研究目的にのみ使用されます。

皆様の本アンケートへのご協力に心より感謝いたします。

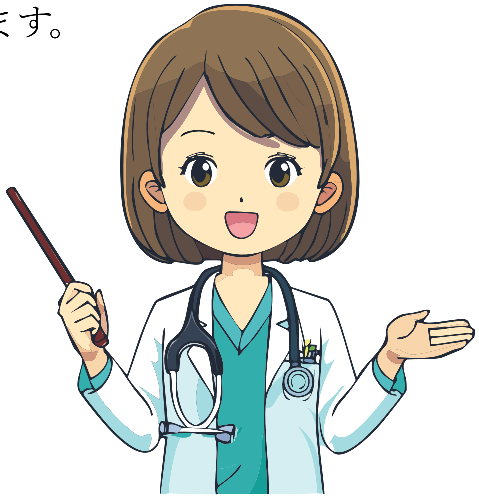

番号：

1.「ODI アンケート」

こちらが１つ目のアンケート「ODI アンケート」です。
全部で１０個の質問があり、２～３分程度で終了します。これについて、ご自身の状況に最も当てはまる回答を選んでマークしてください。
該当しない項目がある場合は、空欄のままにしていただいても構いません。

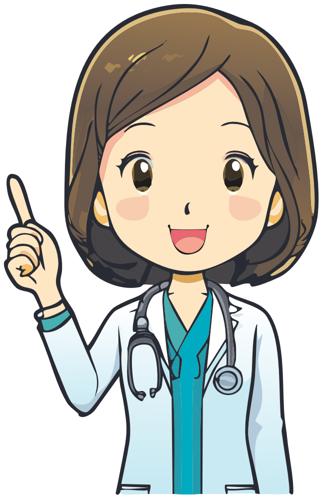

番号：

以下のアンケートに答えてください。これらは、腰の痛み（あるいは足の痛み）が、あなたの日常生活にどのように影響しているのかを知るためのものです。
それぞれの項目の中で、もっともあなたの状態に近いものを選んで○をつけてください。

1：痛みの強さ

(　　)0　今のところ、痛みは全くない
(　　)1　今のところ、痛みはとても軽い
(　　)2　今のところ、中くらいの痛みがある
(　　)3　今のところ、痛みは強い
(　　)4　今のところ、痛みはとても強い
(　　)5　今のところ、想像を絶するほどの痛みがある

２：身の回りのこと（洗顔や着替えなど）

(　　)0　痛みはなく、普通に身の回りのことができる
(　　)1　身の回りのことは普通にできるが、痛みがでる
(　　)2　身の回りのことはひとりでできるが、痛いので時間がかかる
(　　)3　少しの助けは必要だが、身の回りのほとんどのことは、どうにかひとりでできる
(　　)4　身の回りのほとんどのことを、他の人に助けてもらっている
(　　)5　着替えも洗顔もできず、寝たきりである

３：物を持ち上げる

(　　)0　痛みはなく、重い物を持ち上げることができる
(　　)1　重い物を持ち上げられるが、痛みがでる
(　　)2　床にある重い物は痛くて持ち上げられないが、（テーブルの上などにあり）持ちやすくなっていれば、重い物でも持ち上げられる
(　　)3　重い物は痛くて持ち上げられないが、（テーブルの上などにあり）持ちやすくなっていれば、それほど重くない物は持ち上げられる
(　　)4　軽い物しか持ち上げられない
(　　)5　何も持ち上げられないか、持ち運びもできない

番号：

4 ：歩くこと

(　　)0　いくら歩いても痛くない
(　　)1　痛みのため、 1 km 以上歩けない
(　　)2　痛みのため、500m 以上歩けない
(　　)3　痛みのため、100m 以上は歩けない
(　　)4　杖や松葉杖なしでは歩けない
(　　)5　ほとんど部屋で過ごし、歩けない

5 ：座ること

(　　)0　どんな椅子にでも、好きなだけ座っていられる
(　　)1　座り心地の良い椅子であれば、いつまでも座っていられる
(　　)2　痛みのため、 1 時間以上は座っていられない
(　　)3　痛みのため、30 分以上は座っていられない
(　　)4　痛みのため、10 分以上は座っていられない
(　　)5　痛みのため、座ることができない

6 ：立っていること

(　　)0　痛みなく、好きなだけ立っていられる
(　　)1　痛みはあるが、好きなだけ立っていられる
(　　)2　痛みのため、 1 時間以上は立っていられない
(　　)3　痛みのため、30 分以上は立っていられない
(　　)4　痛みのため、10 分以上は立っていられない
(　　)5　痛みのため、立っていることができない

7 ：睡眠

(　　)0　痛くて目を覚ますことはない
(　　)1　ときどき、痛くて目を覚ますことがある
(　　)2　痛みのため、 6 時間以上は眠れない
(　　)3　痛みのため、 4 時間以上は眠れない
(　　)4　痛みのため、 2 時間以上は眠れない
(　　)5　痛みのため、眠ることができない

番号：

８：性生活について（関係あれば）

(　　)0　性生活はいつもどおりで、痛くはない
(　　)1　性生活はいつもどおりだが、痛みがでる
(　　)2　性生活は、ほぼいつもどおりだが、かなり痛む
(　　)3　性生活は、痛みのためにかなり制限される
(　　)4　性生活は、痛みのためにほとんどない
(　　)5　性生活は、痛みのためまったくない

９：社会生活（仕事以外での付き合い）

(　　)0　社会生活はふつうで、痛みはない
(　　)1　社会生活はふつうだが、痛みが増す
(　　)2　スポーツなどのように、カラダを動かすような物をのぞけば、社会生活に大きな影響はない
(　　)3　痛みのため社会生活は制限され、あまり外出しない
(　　)4　痛みのため、社会生活は家の中だけに限られる
(　　)5　痛みのため、社会生活はない

１０：乗り物での移動

(　　)0　痛みなくどこへでも行ける
(　　)1　どこへでも行けるが、痛みがでる
(　　)2　痛みはあるが、２時間程度なら乗り物に乗っていられない
(　　)3　痛みのため、１時間以上は乗っていられない
(　　)4　痛みのため、30 分以上は乗っていられない
(　　)5　痛みのため、病院へ行くとき以外は乗り物には乗れない

番号：

2.「FSS アンケート」

こちらが２つ目のアンケート「ＦＳＳアンケート」です。
全部で１８個の質問があり、３～４分程度で終了します。
もし、当てはまるものがなければ、「**該当なし**」に印を付けてください。

アンケートの後に、２つのアンケートに対する感想を問うアンケートがございます。
こちらは必ずご回答いただきますようお願いいたします。

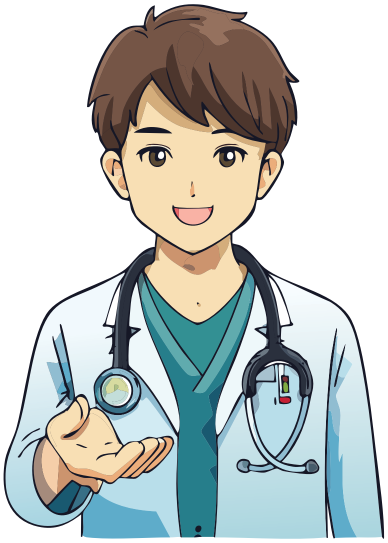

番号：

TOKAI UNIVERSITY

手術後、背骨が気になることはありますか？<br>以下の項目で、当てはまるものにチェックしてください。	全くない	めったにない	時々ある	よくある	いつもある	該当なし
1　仰向けに横になっているとき	o	o	o	o	o	o
2　15 分以上座っているとき	o	o	o	o	o	o
3　15 分以上立っているとき	o	o	o	o	o	o
4　階段を上っているとき	o	o	o	o	o	o
5　自分で身体を洗ったり服を着たりするとき	o	o	o	o	o	o
6　歩いているとき	o	o	o	o	o	o
7　車を運転しているとき	o	o	o	o	o	o

番号：

TOKAI UNIVERSITY

手術後、背骨が気になることはありますか？<br>以下の項目で、当てはまるものにチェックしてください。	全くない	めったにない	時々ある	よくある	いつもある	該当なし
8　家庭での活動をしているとき（例：掃除、庭仕事など）	O	O	O	O	O	O
9　靴下をはいたり靴紐を結んだりするとき	O	O	O	O	O	O
10　床から何かを拾うとき	O	O	O	O	O	O
11　座っているまたは寝ている状態から立ち上がるとき	O	O	O	O	O	O
12　バッグを持ったり、リュックを背負って歩いているとき	O	O	O	O	O	O
13　重い物（10kg 以上）を持ち上げるとき（例：掃除機など）	O	O	O	O	O	O
14　中程度の重さ（ 2kg 以内）の物を 15 分以上持っているとき	O	O	O	O	O	O

番号：

手術後、背骨が気になることはありますか？<br>以下の項目で、当てはまるものにチェックしてください。	全くない	めったにない	時々ある	よくある	いつもある	該当なし
15　手術の傷が見える可能性のある活動	o	o	o	o	o	o
16　パーティーやコンサートなどの社交イベント	o	o	o	o	o	o
17　スポーツ活動	o	o	o	o	o	o
18　セックスや親密な活動	o	o	o	o	o	o

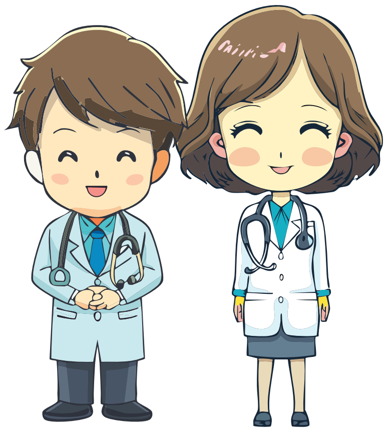

教えてください

年　　齢：　　____ 才

性　　別：　　男性 ・女性 ・ その他

最終学歴：　　小学校 ・中学校 ・高校 ・ 大学

あと 1 ページです!

番号：

TOKAI UNIVERSITY

最後に、FSS アンケートについてのご感想をお聞かせください。

① 1つ目のアンケート(ODI)と比べて、以下ついてどのように感じますか？　(複数回答可)
「答えやすい」・「 変わらない」・「長すぎる」・「質問が足りない」・「質問が難しい」

② 2つ目の(FSS)をどのように評価しますか？
「とても良い」・「良い」・「普通」・「良くない」・「とても良くない」

③ 1つ目のアンケート(ODI)と比べて、脊椎手術後の生活の質に寄り添ったアンケートとなっていましたか？　「はい」・「いいえ」・「意見なし」

④ その他のご意見やコメントがあれば、記載ください：

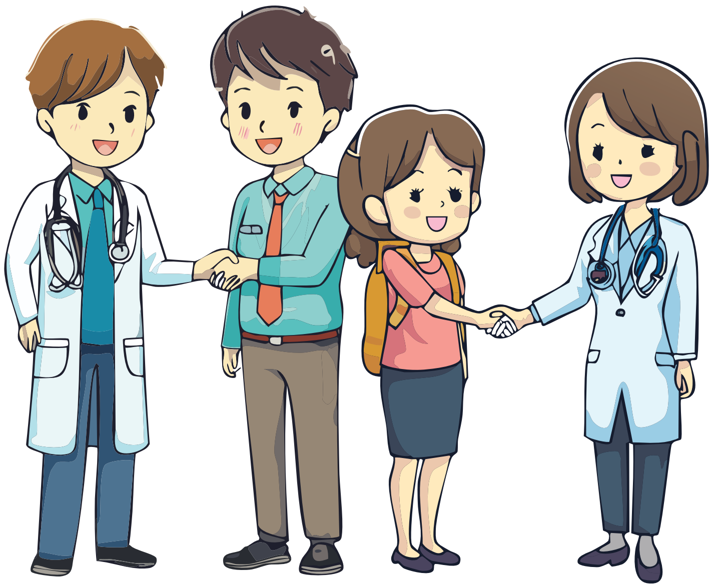

以上でアンケートは終了です。お答えいただき、誠にありがとうございました。皆様のご協力は脊椎固定術の結果をサポートする新しいツールの開発に役立たせていただきます。用紙は、受付にご提出ください。

番号：
