# Supplementary material for: The forgotten spine score: study protocol for a novel patient-centered outcome measure in lumbar spine fusion surgery
Source: Front Surg. 2025 May 22;12:1547829. doi: 10.3389/fsurg.2025.1547829 (PMC12231350; doi:10.3389/fsurg.2025.1547829)
Supplement: Supplementary file 2 [file Datasheet2.pdf]

# Forgotten Spine Score

Benvenuto/a! Questo sondaggio ha l'obiettivo di validare l'utilizzo di un nuovo questionario per valutare i risultati clinici dell'intervento chirurgico di stabilizzazione vertebrale lombare. Nonostante numerose scale siano già state sviluppate a questo scopo, la letteratura riconosce unanimemente in esse un limite: non pongono il paziente al centro dell'indagine.

Poichè nel nostro approccio clinico riteniamo che il paziente sia il protagonista del percorso terapeutico, abbiamo voluto sviluppare un questionario incentrato su come il paziente percepisce il ruolo della chirurgia nella propria vita quotidiana.

Siamo partiti da un concetto semplice: avremo avuto davvero successo solo quando il paziente dimenticherà di essersi operato! La invitiamo pertanto a rispondere a questo nuovo questionario, da noi rinominato "Forgotten Spine Score". Le richiediamo, cortesemente, anche di rispondere ad un'altra scala valutativa, l'Oswestry Disability Index. Ciò ci consentirà di confrontare i due strumenti e ottimizzare ulteriormente il nostro.

Il suo contributo sarà fondamentale per migliorare il questionario e consentire che sia utilizzato in larga scala. I dati verranno raccolti in maniera totalmente anonima.

La ringraziamo in anticipo per averci concesso parte del suo prezioso tempo.

Rimaniamo a disposizione per qualunque chiarimento.

## Sezione 1 - Forgotten Spine Score

| | **Consapevolezza generale**<br>Si accorge che la sua colonna è stabilizzata quando… | Mai | Quasi mai | Talvolta | Spesso | Sempre | Non si applica a me |
|---|---|---|---|---|---|---|---|
| 1 | ...è disteso/a? | O | O | O | O | O | O |
| 2 | ...è seduto/a per più di 15 minuti?? | O | O | O | O | O | O |
| 3 | ...è in piedi per più di 15 minuti? | O | O | O | O | O | O |
| 4 | ...sale le scale? | O | O | O | O | O | O |
| 5 | ...si lava e/o si veste? | O | O | O | O | O | O |
| 6 | ...cammina o fa hiking? | O | O | O | O | O | O |
| 7 | ...guida (o è a bordo di) un'auto? | O | O | O | O | O | O |
| | **Attività di piegamento e torsione**<br>Si accorge che la sua colonna è stabilizzata quando… | Mai | Quasi mai | Talvolta | Spesso | Sempre | Non si applica a me |
| 8 | …svolge le faccende domestiche? (ad es., pulizie, giardinaggio, cura dei bambini) | O | O | O | O | O | O |
| 9 | …si piega per indossare i calzini o allacciare le scarpe? | O | O | O | O | O | O |
| 10 | …prende qualcosa dal pavimento? | O | O | O | O | O | O |
| 11 | …si solleva dalla posizione seduta o distesa? | O | O | O | O | O | O |

| **Sollevamento e trasporto pesi**<br>Si accorge che la sua colonna è stabilizzata quando… | Mai | Quasi mai | Talvolta | Spesso | Sempre | Non si applica a me |
|---|---|---|---|---|---|---|
| 12 …cammina trasportando una borsa o uno zaino? | O | O | O | O | O | O |
| 13 …solleva oggetti pesanti (>10 kg)? (ad es., aspirapolvere, casse d'acqua, bambini, valigie, ecc.) | O | O | O | O | O | O |
| 14 …sta mantenendo o trasportando qualcosa di peso intermedio (>2kg) per più di 15 min? (per esempio, buste della spesa, computer, libri, etc.) | O | O | O | O | O | O |

| **Interazioni sociali**<br>La sua colonna stabilizzata le ha impedito di prender parte in… | Mai | Quasi mai | Talvolta | Spesso | Sempre | Non si applica a me |
|---|---|---|---|---|---|---|
| 15 …attività che potrebbero mettere in mostra le tue cicatrici (ad es. nuoto)? | O | O | O | O | O | O |
| 16 …eventi sociali o feste? | O | O | O | O | O | O |
| 17 …attività sportive? | O | O | O | O | O | O |
| 18 …attività sessuali o intime? | O | O | O | O | O | O |

## Sezione 2 – Oswestry Disability Index

Indice di disabilità di Oswestry
Questo questionario è stato elaborato per darci delle informazioni su quanto i suoi problemi alla schiena (o alla gamba) influenzino la sua capacità di cavarsela nella vita di tutti i giorni. La preghiamo di rispondere a tutte le domande del questionario. Faccia una crocetta su una sola casella per ciascuna domanda, scegliendo quella che più si avvicina a come si sente oggi.

**Sezione 1 – Intensità del dolore**
0 □ Al momento non ho dolore
1 □ Al momento il dolore è molto lieve
2 □ Al momento il dolore è di media intensità
3 □ Al momento il dolore è abbastanza forte
4 □ Al momento il dolore è molto forte
5 □ Al momento il dolore è il massimo immaginabile

**Sezione 2 – Cura personale (lavarsi, vestirsi, ecc.)**
0 □ Riesco a prendermi cura di me stesso/a normalmente senza sentire più dolore del solito
1 □ Riesco a prendermi cura di me stesso/a normalmente ma mi fa molto male
2 □ Mi fa male prendermi cura di me stesso/a e sono lento/a e prudente
3 □ Ho bisogno di un po' di aiuto ma riesco per lo più a prendermi cura di me stesso/a
4 □ Ho bisogno di aiuto ogni giorno in quasi tutti gli aspetti della cura di me stesso/a
5 □ Non mi vesto, mi lavo con difficoltà e sto a letto

**Sezione 3 – Alzare pesi**
0 □ Riesco a sollevare oggetti pesanti senza sentire più dolore del solito
1 □ Riesco a sollevare oggetti pensanti ma sentendo più dolore del solito
2 □ Il dolore mi impedisce di sollevare oggetti pesanti da terra, ma ci riesco se sono posizionati in maniera opportuna, per esempio su un tavolo
3 □ Il dolore mi impedisce di sollevare oggetti pesanti, ma riesco a sollevare oggetti leggeri o di medio peso se sono posizionati in maniera opportuna
4 □ Riesco a sollevare solo oggetti molto leggeri
5 □ Non riesco a sollevare o trasportare assolutamente niente

**Sezione 4 – Camminare**
0 □ Il dolore non mi impedisce di percorrere qualsiasi distanza a piedi
1 □ Il dolore mi impedisce di camminare per più di un chilometro
2 □ Il dolore mi impedisce di camminare per più di 500 metri
3 □ Il dolore mi impedisce di camminare per più di 100 metri
4 □ Riesco a camminare solo con un bastone o delle stampelle
5 □ Sto per lo più a letto e mi trascino per arrivare in bagno

**Sezione 5 – Stare seduto/a**
0 □ Riesco a stare seduto/a su qualsiasi sedia per tutto il tempo che mi va
1 □ Riesco a stare seduto/a sulla mia sedia preferita per tutto il tempo che mi va
2 □ Il dolore mi impedisce di stare seduto/a per più di 1 ora

3 ☐ Il dolore mi impedisce di stare seduto/a per più di mezz'ora
4 ☐ Il dolore mi impedisce di stare seduto/a per più di 10 minuti
5 ☐ Il dolore mi impedisce del tutto di stare seduto/a

**Sezione 6 – Stare in piedi**
0 ☐ Riesco a stare in piedi per tutto il tempo che mi va senza sentire più dolore del solito
1 ☐ Riesco a stare in piedi per tutto il tempo che mi va, ma sentendo più dolore del solito
2 ☐ Il dolore mi impedisce di stare in piedi per più di 1 ora
3 ☐ Il dolore mi impedisce di stare in piedi per più di mezz'ora
4 ☐ Il dolore mi impedisce di stare in piedi per più di 10 minuti
5 ☐ Il dolore mi impedisce del tutto di stare in piedi

**Sezione 7 – Dormire**
0 ☐ Il mio sonno non viene mai disturbato dal dolore
1 ☐ Il mio sonno viene disturbato ogni tanto dal dolore
2 ☐ A causa del dolore dormo meno di 6 ore
3 ☐ A causa del dolore dormo meno di 4 ore
4 ☐ A causa del dolore dormo meno di 2 ore
5 ☐ Il dolore mi impedisce del tutto di dormire

**Sezione 8 – Vita sessuale (se pertinente)**
0 ☐ La mia vita sessuale è normale e non mi provoca più dolore del solito
1 ☐ La mia vita sessuale è normale, ma mi provoca più dolore del solito
2 ☐ La mia vita sessuale è quasi normale, ma mi provoca molto dolore
3 ☐ La mia vita sessuale è fortemente limitata dal dolore
4 ☐ La mia vita sessuale è quasi inesistente a causa del dolore
5 ☐ Il dolore mi impedisce del tutto di avere una vita sessuale

**Sezione 9 – Vita sociale**
0 ☐ La mia vita sociale è normale e non mi provoca più dolore del solito
1 ☐ La mia vita sociale è normale, ma aumenta il livello del dolore
2 ☐ Il dolore non ha effetti significativi sulla mia vita sociale, a parte il fatto di limitare alcuni dei miei interessi che richiedono più energia (ad esempio sport, ecc.)
3 ☐ Il dolore limita la mia vita sociale e non esco così spesso come al solito
4 ☐ Il dolore limita la mia vita sociale alla mia abitazione
5 ☐ Non ho vita sociale a causa del dolore

**Sezione 10 – Viaggiare**
0 ☐ Riesco a viaggiare in ogni luogo senza dolore
1 ☐ Riesco a viaggiare in ogni luogo, ma sentendo più dolore del solito
2 ☐ Mi fa male, ma riesco a viaggiare per più di due ore
3 ☐ Il dolore mi limita a viaggi che durano meno di un'ora
4 ☐ Il dolore mi limita a viaggi brevi e necessari che durano meno di 30 minuti
5 ☐ Il dolore mi impedisce di viaggiare, tranne che per fare le mie cure
